# Noncanonical association of EZH2 with E2F1 promotes tumor proliferation through chromatin remodeling

**DOI:** 10.1038/s12276-025-01603-0

**Published:** 2025-12-19

**Authors:** Mijoung Yoo, Hyeonji Lee, Hyorim Park, Byunghee Kang, Hyo-Min Kim, Tae-Kyung Kim, Tae-Young Roh

**Affiliations:** 1 Department of Life Sciences, Pohang University of Science and Technology (POSTECH), Pohang, Republic of Korea; 2 Department of Life Sciences, Ewha Womans University, Seoul, Republic of Korea; 3 College of Pharmacy, Ewha Womans University, Seoul, Republic of Korea; 4 Sysgenlab Inc., Pohang, Republic of Korea

**Keywords:** Epigenomics, Gene regulation

## Abstract

Enhancer of zeste homolog 2 (EZH2), the catalytic subunit of the polycomb repressive complex 2 (PRC2), which mediates transcriptional repression through histone H3 lysine 27 trimethylation (H3K27me3), is highly expressed in aggressive triple-negative breast cancer (TNBC). However, despite the elevated EZH2 expression, H3K27me3 levels remain unexpectedly low, suggesting a potential noncanonical role for EZH2 in TNBC. Here we demonstrate that EZH2 directly binds to the transcription factor E2F1, and this interaction is critical for modulating chromatin accessibility by disrupting H3K27me3 deposition. This noncanonical function of EZH2, which operates independently of its methyltransferase activity, is linked to enhanced tumor cell proliferation and inhibition of apoptosis. Our findings reveal that EZH2 functions in a chromatin context-dependent manner by cooperating with E2F1 in TNBC, highlighting that the EZH2–E2F1 interaction, independent of PRC2, plays a key role in remodeling chromatin structure and facilitating TNBC proliferation.

## EZH2–E2F1 interaction drives triple-negative breast cancer growth

Breast cancer is a leading cancer type in women, with triple-negative breast cancer (TNBC) being particularly aggressive and hard to treat. This study addresses the role of a protein called EZH2 in TNBC, which is known for its involvement in gene regulation. Here researchers explored how EZH2 functions beyond its usual role in a complex called PRC2. The study used various laboratory techniques to investigate how EZH2 interacts with another protein, E2F1, in TNBC cells. They found that EZH2 and E2F1 work together to regulate the cell cycle and prevent cell death, which helps cancer cells grow. The results showed that EZH2 has a unique function in TNBC that does not rely on its typical role in PRC2. This discovery suggests new ways to target TNBC by disrupting the interaction between EZH2 and E2F1.

This summary was initially drafted using artificial intelligence, then revised and fact-checked by the author.

## Introduction

Breast cancer is the most common cancer in women, accounting for approximately 31% of diagnosed cancer, and its incidence has been steadily increasing by approximately 0.5% annually worldwide^1^. It is typically classified according to the expression levels of estrogen receptor (ER), progesterone receptor (PR) and human epidermal growth factor receptor 2 (HER2)^2^. Each class of breast cancer has distinct genetic and epigenetic abnormalities, such as somatic mutations and DNA methylation^3,4^. Therefore, comprehensive investigations on breast cancer have been conducted to dissect its heterogeneous landscape and underlying key biological mechanisms. In particular, triple-negative breast cancer (TNBC; ER, PR and HER negative (ER^−^PR^−^HER^−^)), a highly aggressive breast cancer subtype lacking ER, PR and HER2, is difficult to treat owing to the absence of these receptors^5^. TNBC exhibits high cellular heterogeneity, with certain subtypes having cancer stem cell-like characteristics that contribute to aggressive tendencies and drug resistance. In addition, the tumor microenvironment of TNBC is highly diverse, which further promotes tumor progression and metastasis^6^. Given the poor prognosis, high rate of drug resistance and limited treatment options for TNBC, epigenetic reprogramming has been recognized as a critical driver of tumorigenesis^7^. Aberrant epigenetic modifications such as histone modifications, DNA methylation and chromatin remodeling contribute to the deregulation of oncogenes and tumor suppressor genes. Consequently, the exploration of epigenetic alterations in cancer has garnered considerable attention, opening new avenues for targeted therapies. The development of epigenetic therapies, or epi-drugs such as DNA methyltransferase inhibitors and histone deacetylase inhibitors, aims to reverse these epigenetic aberrations, offering potential new treatment strategies for TNBC and other malignancies^8^.

EZH2, a catalytic subunit of polycomb repressive complex 2 (PRC2), mediates the trimethylation of histone H3 at lysine 27 (H3K27me3) for transcriptional repression, which is regarded as the canonical function^9,10^. PRC2 also functions as an epigenetic regulator or a histone modifier across various tissues and during developmental processes, primarily responsible for the precise repression of gene expression^11,12^. Aberrant activation of PRC2 facilitates tumor progression and metastasis by silencing tumor suppressor genes^13,14^, prompting the development of multiple PRC2 inhibitors for cancer therapy^15,16^. The transcription of the PRC2 target genes is controlled by growth factors through the pRB–E2F pathway which is characterized in cell proliferation and tumorigenesis^17^. However, EZH2 free from other components of PRC2 might have a noncanonical function for cancer progression^18^. In particular, TNBC, characterized by low levels of H3K27me3 despite high EZH2 expression, could be a model cancer to investigate the noncanonical functions of EZH2 owing to this unique characteristic^19^. Previous studies reported that EZH2 facilitates invasion and metastasis in TNBC by promoting the expression of matrix metalloproteinase genes (MMPs) and NF-kB target genes with RELB^20,21^ and FOXM1 (ref. ^22^). Even though activated genes by EZH2 in TNBC are closely associated with the cell cycle, the noncanonical mechanisms of EZH2 for cell cycle progression and its specific contexts remain unclear.

Here, our study elucidated the noncanonical mechanisms of EZH2 in TNBC through cellular and multiomics approaches together with molecular and cellular assays. EZH2 interacts with E2F1 at its SANT2 domain to regulate cell cycle progression through chromatin remodeling in TNBC. This interaction contributes to chromatin remodeling but does not affect PRC2 activity.

## Materials and methods

### Analysis with TCGA datasets

Transcriptome profiles from primary breast carcinoma (BRCA) datasets (*n* = 1229) were downloaded from the National Cancer Institute Genomic Data Commons Portal (https://portal.gdc.cancer.gov/). The normalized transcripts per million (TPM) values were scaled to log_10_ and displayed in a box plot to compare the differences between the two groups. The significance of differences between groups was estimated using the Mann–Whitney *U* Test after assessing data for normal distribution and homogeneity of variance. The relative protein levels were quantified using data from the The Cancer Genome Atlas (TCGA) Breast Cancer Proteomics Study (PDC000173) (*n* = 105) in the Proteomic Data Commons (PDC).

### Cell culture and drug treatment

The MDA-MB-231, MCF7, and Hs578T cell lines were obtained from the Korean Cell Line Bank. The HEK293T cell line was purchased from the American Type Culture Collection. Both cell lines were cultured in Dulbecco’s modified Eagle medium (DMEM) supplemented with 10% fetal bovine serum (FBS) and 1% penicillin/streptomycin at 37 °C in a humidified atmosphere with 5% CO_2_. For the drug treatment, cells were exposed to GSK126 (Tocris Bioscience, 6790) for 72 h.

### Gene knockdown

The pLKO.1-TRC cloning vector (Addgene, 10878) was used to generate the short hairpin RNA (shRNA) construct. The shRNA sequences are detailed in Supplementary Table 1. The construction of the shRNA was verified through DNA sequencing. For the production of lentivirus, HEK293T cells were cotransfected with the lentiviral plasmid encoding the shRNA construct and the lentiviral packaging plasmids (pMD2.G and pCMV.dR 8.74) using polyethylenimine (Polyscience, 23966). Lentiviruses were collected at 48- and 72-h post-transfection and then filtered through a 0.45-μm filter. For lentiviral transduction, 0.2 million cells were plated in a 6-well plate 1 day before transfection. Cells with approximately 70–80% confluency were transduced with the lentivirus. At 24 h after transduction, cells were selected with puromycin (2 μg/ml, Sigma-Aldrich, P8833) for 72 h.

### Protein overexpression analysis

The pCMVHA hEZH2 vector (Addgene, 24230) and the pCMVHA hE2F1 vector (Addgene, 24225) were acquired from Addgene. The *E2F1* sequence was inserted into the pCS2FLAG vector (Addgene, 16331). For transfection, cells with 70–80% confluency were transfected with 2 μg of DNA using Lipofectamine 3000 (Invitrogen, L3000001) according to the manufacturer’s instructions. After 72 h, protein expression was evaluated by western blot and further experiments were conducted.

### Mass spectrometry analysis

Immunoprecipitated samples were resolved by SDS–PAGE, and the entire gel lanes were excised and subjected to in-gel tryptic digestion. For EZH2- and E2F1-associated protein complexes, the digested peptides were analyzed with timsTOF Pro 2 (Bruker Daltonics) and Orbitrap Exploris 480 (Thermo Fisher Scientific) mass spectrometers, respectively. Peptides were identified using the UniProt database (UniProtKB/Swiss-Prot, *Homo sapiens*, Swissprot.fasta (2024_07_26), 20,436 sequences). The protein–protein interaction network was visualized using Cytoscape (v3.10.3). The identified proteins are listed in Supplementary Table 2.

### Cell proliferation assay

After 96 h of transduction, an equal number of cells (1000 cells/well) were seeded in each well of 12-well plates in triplicate. Every 24 h, living cells were stained with trypan blue and counted by Countess III automated cell counter (Invitrogen).

### Apoptosis and cell cycle analysis

Cells transduced with the lentivirus were collected at 72 h post-transduction and washed with ice-cold PBS. For the apoptosis assay, cells were stained with the FITC Annexin V Apoptosis Detection Kit with propidium iodide (PI) (BioLegend, 640914) according to the manufacturer’s instructions. For the cell cycle analysis, cells were fixed with ice-cold 70% ethanol overnight at 4 °C. Fixed cells were washed and permeabilized in PBS containing 0.25% Triton X-100 on ice for 15 min. The cells were stained with PBS containing 10 μg/ml RNase A (Thermo Scientific, EN0531) and 20 μg/ml PI (BioLegend, 640914) at room temperature in the dark for 30 min. Finally, cells were analyzed by flow cytometry.

### Immunofluorescence staining

Cells were seeded in 4-well chamber slides (SPL Life Science, 30504) and fixed with 4% paraformaldehyde (Thermo Fisher Scientific, 28906). After three washes with cold PBS, the fixed cells were incubated in PBS with 0.4% Triton X-100 at 25 °C for 30 min. Subsequently, the cells were blocked in PBS with 5% bovine serum albumin at 25 °C for 1 h followed by overnight incubation at 4 °C with the primary antibodies. The next day, the cells were washed five times with PBS for 5 min each wash and incubated with secondary antibodies at 25 °C for 1 h. The cells were washed a further five times with PBS and incubated with Hoechst 33342 (10 μg/ml in PBS) for 20 min at room temperature. Antibody information is provided in Supplementary Table 3.

### Co-IP and western blot

For co-immunoprecipitation (co-IP), the protein was isolated from cells with ice-cold IP lysis buffer (150 mM NaCl, 10 mM Tris pH 7.4, 1% Nonidet-P40 and 0.1% sodium deoxycholate) supplemented with a protease inhibitor cocktail (Roche, 11697498001) and 1 mM phenylmethylsulfonyl fluoride. The cell lysate was centrifuged at 16,000*g* for 10 min and the supernatant was precleared with prewashed Dynabead Protein G (Thermo Fisher Scientific, 10003D) for 1 h at 4 °C. The precleared protein was mixed with antibody-bound Dynabead Protein G and incubated overnight at 4 °C. The bead was washed three times with IP washing buffer (150 mM NaCl, 10 mM Tris pH 7.4, 1 mM EDTA and 1% Triton X-100). The eluted proteins were immunoblotted using the antibodies listed in Supplementary Table 3. The proteins were detected using SuperSignal West Blot Pico PLUS (Thermo Fisher Scientific, A43840).

### Histone isolation

Cells were collected and washed by ice-cold PBS. Following washing, the cell pellet was lysed with Triton extraction buffer (PBS containing 0.5% Triton X-100 (v/v), 2 mM PMSF and 0.02% (w/v) NaN_3_) for 10 min on ice. The histone was eluted in 0.2 N HCl by rotation overnight at 4 °C. Histone levels were measured by western blot.

### ChIP-seq and data analysis

Chromatin immunoprecipitation followed by sequencing (ChIP-seq) was conducted as previously described^23,24^. Briefly, the chromatin was fragmented by sonication for transcription factor ChIP or micrococcal nuclease (MNase) (Sigma-Aldrich, N3755) for histone modification ChIP. The antibody information is provided in Supplementary Table 3. ChIP-seq libraries were prepared using the ACCEL-NGS 2S Plus DNA Library Kit (Swift Bioscience, 28096) following the manufacturer’s instructions. All sequencing libraries underwent 150-bp paired-end sequencing on an Illumina NextSeq 1000 or NovaSeq 6000 platform.

Sequencing quality was assessed by FastQC v0.11.5 (ref. ^25^) and adapter sequences were removed using cutadapt (v4.5; −e 0.2)^26^. Reads filtered by Picard and Samtools were mapped to the human genome (ensGene_108.GRCh38) using Bowtie2 (v2.5.2; -X2000 --end-to-end)^27^. Peak calling was performed by MACS2 (v2.2.9.1; -q 0.01 --broad-cutoff 0.1 with the broad option for histone peaks)^28^. Normalized ChIP-seq signals were visualized in Integrative Genomics Viewer (IGV, Broad Institute). Heat maps for ChIP-seq were generated using deeptools (v3.5.4) computeMatrix and plotHeatmap functions^29^. Differential binding regions were discovered using DESeq2 and bedtools^30,31^. The differentially binding regions were visualized in the volcano plot using R packages. Motif discovery analysis for EZH2 and E2F1 targeted regions was conducted using hypergeometric optimization of motif enrichment (HOMER)^32,33^.

### RNA-seq and data analysis

The mRNA-seq libraries were made using the NEBNext Ultra II Directional RNA Library Prep Kit (NEB, E7760S) and the mRNA Magnetic Isolation Module (NEB, E7490S).

Filtered sequencing reads were mapped to the human genome (hg38.ensGene_108) using STAR (v2.7.11a)^34^. Transcript abundance was estimated by RSEM (–estimate-rspd) and differential gene expression by DESeq2 (v1.42.0) in the R environment. The gene set enrichment analysis (GSEA) was performed using the Molecular Signatures Database (MSigDB) v7.2 and GSEA-4.3.3 with the following options: -metric Signal2Noise -permute gene set^35,36^. Gene Ontology (GO) analysis was conducted with the Enrichr web tool^37^ using the Transcriptional Regulatory Relationships Unraveled by Sentence-based Text mining (TRRSUT) databases for upstream transcription factors^38^ and the BioPlanet 2019 databases for biological processes^39^.

### ATAC-seq and data analysis

The Assay for Transposase-Accessible Chromatin (ATAC)-seq was conducted as previously described^23,40^. The DNA was purified using the DNA Clean & Concentrator-5 Kit (Zymo Research, D4003) and then amplified using the NEBNext High-Fidelity 2× PCR Master Mix (NEB, M0541S) for seven cycles. All sequencing reads were aligned to the human genome using Bowtie2 with parameters: ‘-k 4 – end-to–end’. To account for the Tn5 enzyme’s cutting position, ATAC-seq reads were shifted by +4 bp for the positive strand and −5 bp for the negative strand before peak calling. ATAC reads were normalized by counts per million.

## Results

### EZH2 shows a PRC2-independent function in TNBC

**Fig. 1:**
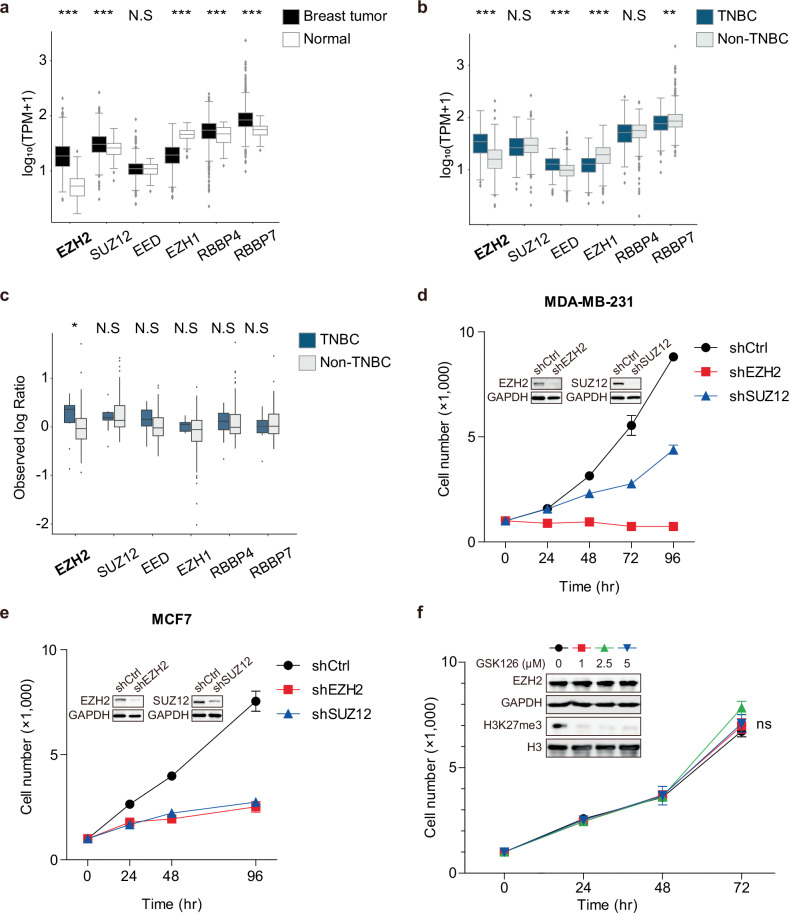
EZH2 shows a noncanonical function in TNBC. **a** The expression levels of PRC2 components in human breast tumors (*n* = 1116) and normal breast tissues (*n* = 113) from the TCGA BRCA dataset. **b** The expression of PRC2 components in TNBC (*n* = 115) and non-TNBC (*n* = 604) groups from the TCGA BRCA dataset. All data represent mean ± s.e.m.; **P ≤ 0.01, ***P ≤ 0.001, N.S., not significant. **c** The relative protein levels of PRC2 components in TNBC (*n* = 11) and non-TNBC (*n* = 94) groups using isobaric tags for relative and absolute quantitation-based quantification from the TCGA Breast Cancer Proteomics Study dataset. TNBC groups were selected based on low expression levels of ERBB2, ESR1 and PGR, defined as less than −1. To assess group differences in protein expression, the Wilcoxon rank-sum test was applied. All data represent mean ± s.e.m.; *P ≤ 0.05. **d** The effect of EZH2 or SUZ12 knockdown on the cell growth in MDA-MB-231 cells. The inset shows the protein levels of EZH2 and SUZ12. **e** The effect of EZH2 or SUZ12 knockdown on the cell growth in MCF7 cells. The inset shows the protein levels of EZH2 and SUZ12. shCtrl indicates cells transduced with a control vector. **f** The effect of GSK126 (1–5 μM) on the cell growth in MDA-MB-231 cells (*n* = 3; mean ± s.d.; unpaired two-tailed Student’s *t*-test). The inset shows the dose-dependent effect on the protein levels of EZH2 and H3K27me3.

EZH2 exhibits PRC2-independent functions in TNBC and is uniquely upregulated at both the transcript and protein levels, correlating with poor prognosis. To assess the distinct characteristics of EZH2 with other PRC2 components in breast cancer, the expression levels of PRC2 components (EZH2, SUZ12, EED, EZH1, RBBP4, and RBBP7) were evaluated using TCGA datasets. All PRC2 components, except for *EED* and *EZH1*, were significantly enriched in breast tumors compared to normal tissues (Fig. 1a). While *SUZ12*, *RBBP4* and *RBBP7* were highly expressed in breast tumors, their transcription levels were either comparable to or lower in TNBC groups compared to non-TNBC groups (Fig. 1b). Consistent with transcriptomic data, EZH2 and EED proteins were more abundantly expressed in TNBC groups compared to non-TNBC samples (Fig. 1c). *EZH2* was the only gene that showed significantly elevated expression at both the transcriptional and translational levels in TNBC compared to non-TNBC subtypes, as well as in breast tumors relative to normal tissues. To determine the clinical relevance of these findings, patient survival data from TCGA were further analyzed. High *EZH2* expression was associated with poorer survival in TNBC subtypes, whereas *SUZ12* expression was not (Supplementary Fig. 1a,b).

We further explored whether EZH2 exhibits functions distinct from its canonical role as a component of PRC2 in TNBC cell proliferation. The functional roles of two major PRC2 components, EZH2 and SUZ12, were investigated in the TNBC cell lines (MDA-MB-231 and Hs578T) and ER^+^ cell line, MCF7. The knockdown of EZH2 suppressed almost completely cell growth in MDA-MB-231 and Hs578T, but that of SUZ12 showed a decreased cell growth rate (Fig. 1d and Supplementary Fig. 1c). In contrast, the knockdown of either EZH2 or SUZ12 led to a remarkable and comparable reduction in MCF7 cell proliferation (Fig. 1e). This suggests that EZH2 functions not only as a PRC2 component but also through a noncanonical mechanism for the cell proliferation of TNBC. The methyltransferase activity of EZH2 was blocked by GSK126, an inhibitor that competes for its *S*-adenosylmethionine binding site. GSK126 treatment at various concentrations dramatically reduced H3K27me3 levels but had no effect on the growth rate of TNBC cells (Fig. 1f). The inhibitor treatment effectively reduced global H3K27me3 levels without changing the expression levels of EZH2 and SUZ12 (Supplementary Fig. 1d).

### EZH2 interacts with E2F1 for the noncanonical function

**Fig. 2:**
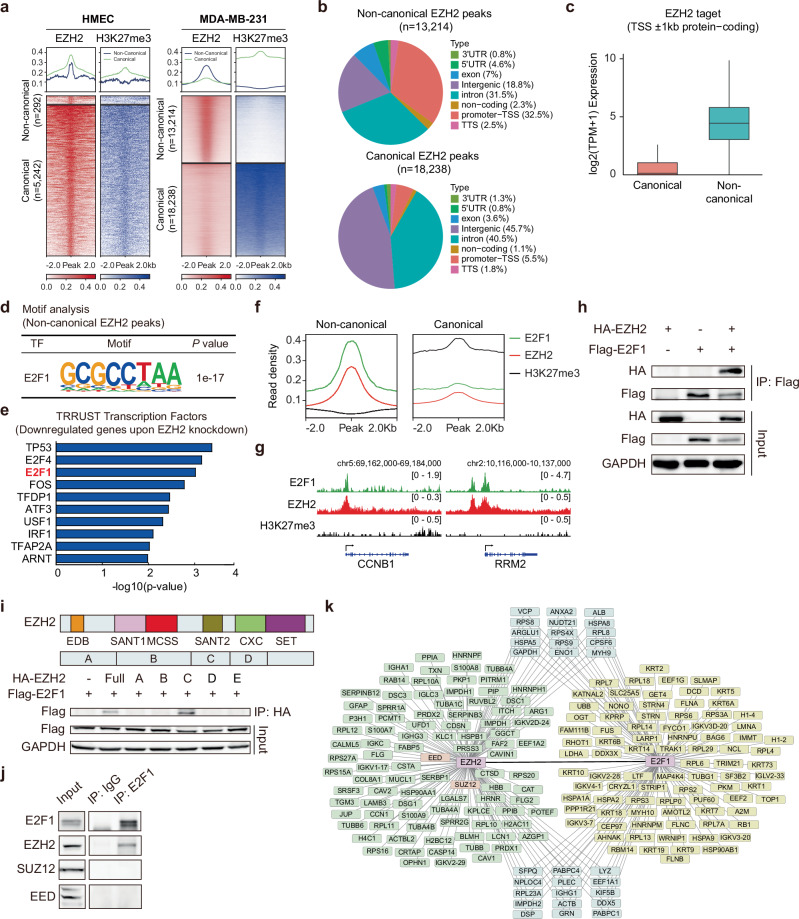
The association of EZH2 with E2F1 is required for the noncanonical function. **a** The enrichment profiles of EZH2 and H3K27me3 for EZH2^+^H3K27me3^−^ (noncanonical) and EZH2^+^H3K27me3^+^ (canonical) regions in HMEC and MDA-MB-231 cells, showing the read density for enrichment levels (top) and heat map (bottom) **b** Genome-wide distribution of EZH2 and H3K27me3 peaks for the EZH2^+^H3K27me3^−^ regions (top, noncanonical regions) and EZH2^+^H3K27me3^+^ (bottom, canonical regions). **c** Gene expression levels of canonical (*n* = 812) and noncanonical EZH2 target genes (*n* = 3,653). Gene expression levels in MDA-MB-231 cells were calculated in TPM from RNA-seq data. **d** A highly enriched transcription factor-binding motif identified by the motif analysis using noncanonical EZH2 target regions. **e** Transcription factors related to downregulated genes upon EZH2 knockdown (*n* = 1391). **f** The average enrichment patterns of EZH2, E2F1 and H3K27me3 for noncanonical (left) and canonical (right) regions centered at EZH2 peaks. **g** Colocalization of EZH2 and E2F1 and distribution of H3K27me3 at the *CCNB1* and *RRM2* locus. **h** The physical interaction between EZH2 and E2F1. **i** Domain organization of EZH2 and its truncated forms (top) and the physical interactions between ectopically expressed Flag–E2F1 in HEK293T cells and full-length and truncated forms of HA–EZH2 (bottom). **j** The physical interaction between E2F1 and PRC2 components. **k** A protein–protein interaction network of top 10% of proteins identified from EZH2 and E2F1 interactomes.

To dissect the PRC2-independent function of EZH2, the enrichment profiles of EZH2 and H3K27me3 were analyzed using ChIP-seq datasets in human mammary epithelial cells (HMECs)^41^ and MDA-MB-231 cells^20,42^. The canonical (EZH2^+^/H3K27me3^+^) and noncanonical peaks (EZH2^+^/H3K27me3^−^) were classified (Fig. 2a). Only a small fraction of EZH2 target regions (292 of 5,534; 5.3%) were noncanonical peaks in HMECs, in contrast to 13,214 of 31,452 (42%) in MDA-MB-231 cells. The noncanonical peaks were mostly positioned at promoter transcription start site (TSS) regions (32.5%) and intron regions (31.5%), whereas the canonical ones belonged to intergenic (45.7%) and intron regions (40.5%) (Fig. 2b). Canonical EZH2 target genes were expressed at low levels, while noncanonical targets showed markedly higher expression, indicating a repressive chromatin state and a transcriptionally active state, respectively (Fig. 2c).

Since many noncanonical peaks in promoter TSS regions suggest a potential role in transcriptional regulation, transcription factor-binding motif analysis was performed on noncanonical EZH2 peaks. The binding motif of E2F1, including those of FOSL2, CTCF and BARHL1, was overrepresented in EZH2-associated regions (Fig. 2d and Supplementary Fig. 2a). Differentially expressed genes were identified by comparative RNA-seq analysis before and after EZH2 knockdown, and 952 upregulated genes by EZH2 knockdown were mostly associated with H3K27me3 histone marks (Supplementary Fig. 2b,c). Additionally, to identify potential transcription factors linked with EZH2, the 1,391 downregulated genes were analyzed. Transcriptional regulatory network analysis revealed the top ten transcription factors: TP53, E2F4, E2F1, FOS, TFDP1, ATF3, USF1, IRF1, TFAP2A and ARNT (Fig. 2e).

Remarkably, E2F1 was the only transcription factor recognized as being potentially associated with EZH2 in both transcription factor-binding motif and transcriptional regulatory network analyses. E2F1 ChIP-seq analysis validated that E2F1 peaks colocalized with EZH2 peaks at noncanonical regions lacking H3K27me3 marks (Fig. 2f), which was exemplified by the profiles at the promoter regions of *CCNB1* (cyclin B1) and *RRM2* (ribonucleotide reductase regulatory subunit M2) (Fig. 2g). GSEA also confirmed that EZH2 knockdown reduced the expression of E2F target genes (Supplementary Fig. 2d). E2F1 exhibited higher expression levels in breast cancers compared to normal tissues (Supplementary Fig. 2e), as well as in TNBC groups in comparison to non-TNBC groups (Supplementary Fig. 2f).

The physical interaction between EZH2 and E2F1 proteins was assessed by a co-IP assay. HA–EZH2 and Flag–E2F1 proteins were overexpressed individually or together in MDA-MB-231 cells verifying the direct binding of EZH2 to E2F1 (Fig. 2h). To characterize the E2F1 binding site on EZH2 domains, the full length of the *EZH2* gene was dissected by domains. The SANT2 domain of EZH2 interacted with E2F1 (Fig. 2i). The SANT2 domain is known to be essential for the interaction of EZH2 with SUZ12, a key component of the PRC2 complex^43^. Core PRC2 components such as SUZ12 and EED, unlike EZH2, did not interact with E2F1 (Fig. 2j).

The 1261 EZH2-associated interacting proteins were identified and involved in multiple biological processes including cytoplasmic translation, translation, gene expression and so on. Canonical PRC2 components such as SUZ12 and EED were detected, supporting the specificity and reproducibility of the assay. Similarly, analysis of E2F1-associated proteins revealed 1166 interacting partners, which were also involved in functions such as mRNA splicing, cytoplasmic translation and gene expression. A protein–protein interaction network was generated based on the top 10% of ranked interactors (Fig. 2k). Among these datasets, 627 proteins were identified as common interacting partners of EZH2 and E2F1. Notably, E2F1 was the only interacting transcription factor obtained from mass spectrometry analysis, which is consistent with the potential binding candidate recognized by transcriptional regulatory network analysis using downregulated genes upon EZH2 knockdown (Fig. 2e) and by motif analysis using noncanonical EZH2 targets (Supplementary Fig. 2a).

Taken together, these results indicate that EZH2 interacts directly with the transcription factor E2F1 at chromatin regions lacking H3K27me3 marks in TNBC.

### The EZH2–E2F1 complex regulates cellular physiology

**Fig. 3:**
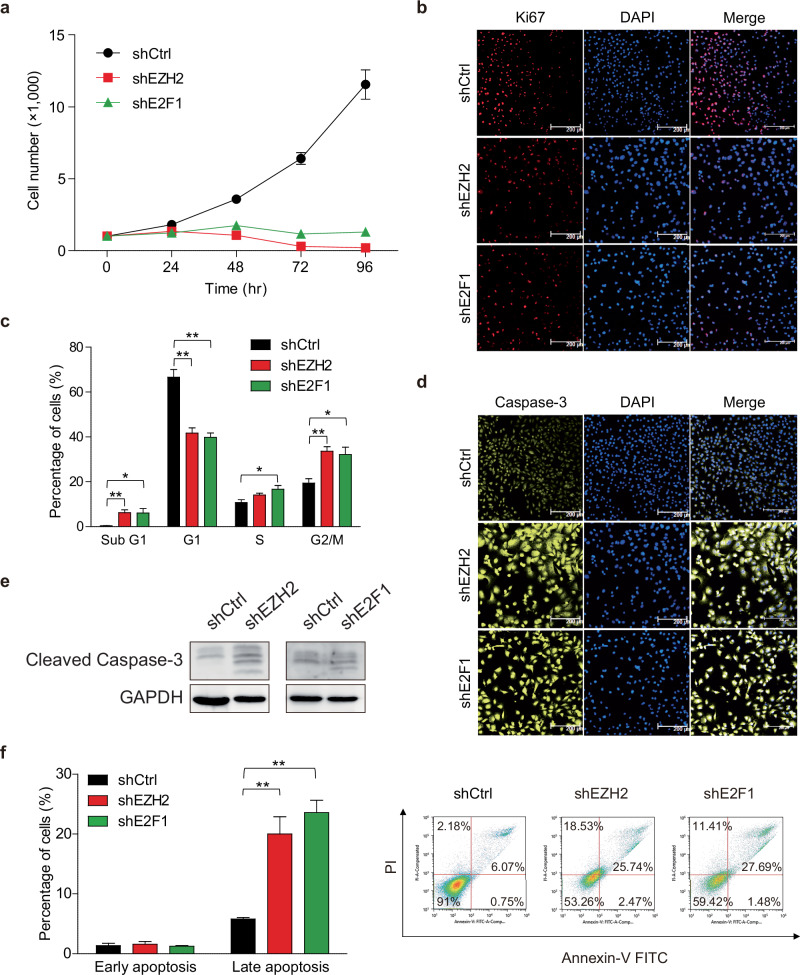
EZH2 and E2F1 play roles in cellular functions. **a** The effect of cell growth under EZH2 or E2F1 knockdown (*n* = 3; mean ± s.d.; unpaired two-tailed Student’s *t*-test). **b** Regulation of cell proliferation by shEZH2 or shE2F1 (scale bar, 200 μm). **c** Cell cycle analysis by flow cytometry (*n* = 3; mean ± s.d.; unpaired two-tailed Student’s *t*-test). **d** The effect of shEZH2 or shE2F1 on cell apoptosis (scale bar, 200 μm). **e** The effect of shEZH2 or shE2F1 on cleaved caspase-3 level. **f** Early and late apoptotic cells measured by flow cytometry (*n* = 3; mean ± s.d.; unpaired two-tailed Student’s *t*-test). One representative profile is shown (right). *P < 0.05, **P < 0.01 and ***P < 0.005. shCtrl indicates cells transduced with a control vector.

Since EZH2 and E2F1 form a complex independent of PRC2, their physiological functions were examined by cell proliferation assay and cell cycle analysis. Individual downregulation of EZH2 or E2F1 notably reduced cell growth (Fig. 3a). Moreover, knockdown of EZH2 or E2F1 led to a reduction in cell proliferation, as indicated by decreased Ki-67 expression, a marker of cellular proliferation (Fig. 3b). Since E2F1 is a well-characterized transcription factor involved in cell cycle regulation, apoptosis and DNA damage response^44^, the impacts of EZH2 and E2F1 on cell cycle dynamics were investigated. Both EZH2 and E2F1 affected the cell number in the G1 phase (for EZH2 knockdown, 24.9% decreased; for E2F1 knockdown, 26.8% decreased), the sub-G1 (for EZH2 knockdown, 5.8% increased; for E2F1 knockdown, 5.7% increased) and the G2/M phases (for EZH2 knockdown, 14.2% increased; for E2F1 knockdown, 12.7% increased). Owing to the loss of function of EZH2 or E2F1, cells were not progressing normally and accumulated in the G2/M phase (Fig. 3c).

Given that EZH2 or E2F1 knockdown remarkably reduced cell proliferation and increased the cell population in the sub-G1 phase, the relationship of EZH2 and E2F1 with apoptosis was further examined. As predicted, perturbation in the expression of EZH2 or E2F1 led to elevated caspase-3 protein expression, suggesting enhanced activation of apoptotic pathways (Fig. 3d). To provide a more direct and functionally relevant indicator of apoptosis, we additionally performed western blot analysis for cleaved caspase-3. This result confirmed that knockdown of EZH2 or E2F1 led to elevated levels of cleaved caspase-3, indicating activation of the apoptotic cascade (Fig. 3e). Assays using annexin V and PI staining confirmed increased late apoptosis upon knockdown of EZH2 or E2F1 (Fig. 3f). Collectively, these results suggest that the EZH2–E2F1 complex contributes to the regulation of cell cycle progression and programmed cell death in TNBC.

### EZH2 and E2F1 change the chromatin accessibility and gene expression programs

To determine whether the EZH2–E2F1 complex coordinately regulates gene expression and chromatin accessibility in TNBC, we investigated their shared genomic targets and transcriptional effects.

**Fig. 4:**
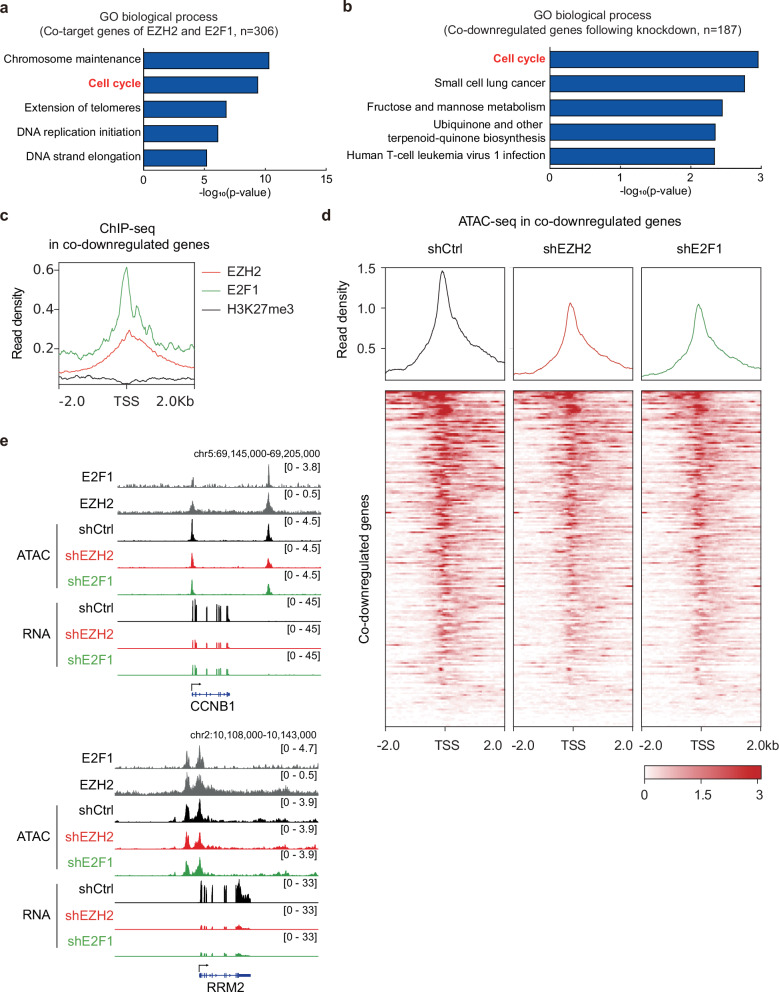
Downstream genes of EZH2 and E2F1 are regulated by changes in chromatin accessibility. **a** GO analysis for biological processes using common target genes of EZH2 and E2F1 (*n* = 306). **b** GO analysis for the 187 co-downregulated genes. **c** Average ChIP-seq profiles of EZH2, E2F1 and H3K27me3 around the TSS regions of the co-downregulated genes. **d** The effect of shEZH2 or shE2F1 in chromatin accessibility around the TSS regions of the co-downregulated genes. **e** The transcription levels and chromatin accessibility by shEZH2 and shE2F1 at the *CCNB1* and *RRM2* locus. shCtrl indicates cells transduced with a control vector.

Target genes regulated by EZH2 and E2F1 were determined by ChIP-seq analysis. Most EZH2 target genes did not overlap with E2F1 target genes. About 38% of the 803 E2F1 target genes coincided with EZH2 target genes. GO analysis with these 306 common genes was performed to define which biological pathways were regulated by the EZH2–E2F1 complex. Cotarget genes of EZH2 and E2F1 were predominantly associated with chromosome maintenance, cell cycle, extension of telomeres, DNA replication initiation and DNA strand elongation (Fig. 4a).

To explore the regulatory function of EZH2 and E2F1, the expression of both EZH2 and E2F1 were markedly suppressed by a knockdown experiment (Supplementary Fig. 2b and 3a) and their consequent gene expression patterns were compared. Upon EZH2 knockdown, 952 genes were upregulated and 1391 genes were downregulated (Supplementary Fig. 3b, left). E2F1 depletion led to the upregulation of 642 genes and the downregulation of 792 genes (Supplementary Fig 3b, right). The 187 commonly downregulated genes were linked to cell cycle, small cell lung cancer and sugar metabolism confirmed by GO analysis (Fig. 4b). A total of 207 co-upregulated genes were associated with neutrophil extracellular trap formation and viral carcinogenesis (Supplementary Fig. 3c,d). As expected, the promoter regions of these commonly downregulated genes were occupied by both EZH2 and E2F1 (Fig. 4c). Specifically, cell cycle-promoting genes such as *CDC20*, *CCNB1*, *PLK1* and *E2F5* were affected by shRNA treatment (Supplementary Fig. 3e). The expression of these cell cycle-related genes was significantly higher in TNBC groups compared to non-TNBC groups (Supplementary Fig. 3f).

Given that chromatin accessibility directly influences gene expression by enabling transcription factors to bind to DNA, ATAC-seq was used to investigate the effects of EZH2 and E2F1 perturbation on chromatin accessibility. Knockdown of EZH2 induced global changes in chromatin accessibility, increasing accessibility in 2320 regions and decreasing it in 978 regions (Supplementary Fig. 4a). E2F1 depletion increased chromatin accessibility in 5212 regions while decreasing it in 599 regions (Supplementary Fig. 4b). When the expression of either EZH2 or E2F1 was inhibited, the chromatin accessibility was decreased at the promoter regions of co-downregulated genes, indicating that chromatin state changes induced by the EZH2–E2F1 complex led to transcriptional alteration of their target genes (Fig. 4d). Specifically, the promoter regions of *CCNB1* and *RRM2*, identified as targets of EZH2–E2F1, exhibited reduced chromatin accessibility and transcription level after their knockdown (Fig. 4e). EZH2 and E2F1 knockdown reduced chromatin accessibility at noncanonical target regions of EZH2 but did not affect chromatin status at canonical target regions of EZH2. (Supplementary Fig. 4c). Altogether, EZH2 and E2F1 act as a unique chromatin regulator to promote the expression of cell cycle-related genes in TNBC, diverging from their conventional roles as a methyltransferase and a transcription factor, respectively.

### E2F1 provides the molecular basis for a novel EZH2 function beyond PRC2-dependent chromatin repression

**Fig. 5:**
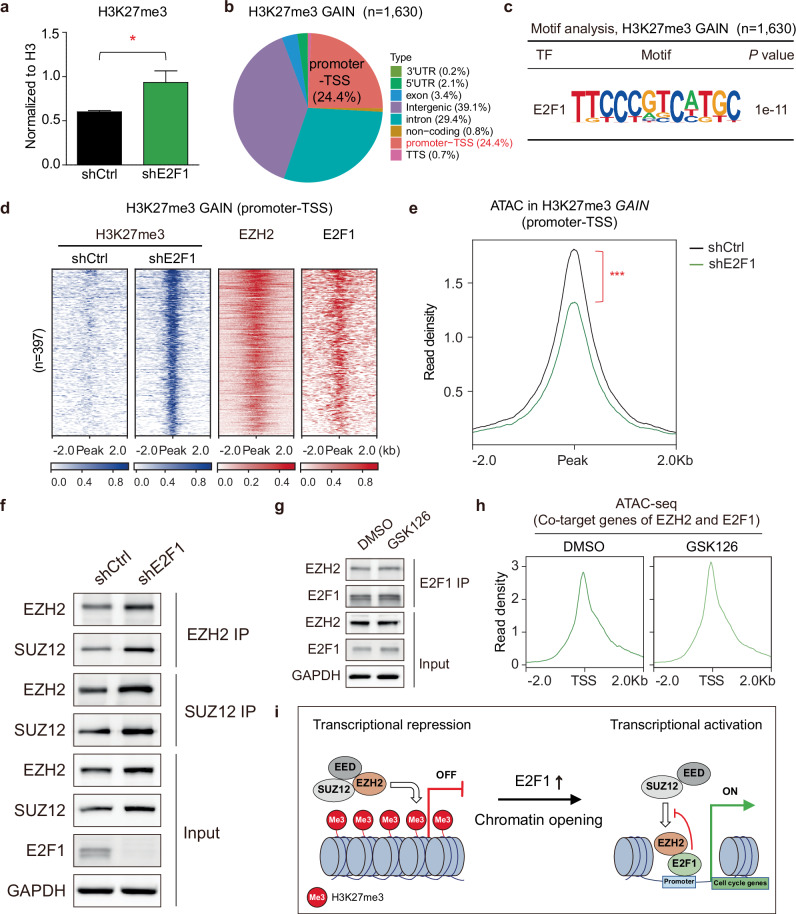
The molecular mechanism of noncanonical functions of EZH2 is associated with E2F1. **a** Global H3K27me3 levels upon E2F1 knockdown (*n* = 3; mean ± s.d.; unpaired two-tailed Student’s *t*-test; *P < 0.05). **b** Genome-wide distribution of H3K27me3 gain regions (*n* = 1,630) by shE2F1. UTR, untranslated region. **c** A highly enriched transcription factor-binding motif identified at H3K27me3 gain regions. **d** Enrichment profiles of EZH2 and E2F1 at the H3K27me3 gain regions induced by shE2F1. **e** Chromatin accessibility around H3K27me3 gain peaks at the TSS promoter region upon shE2F1 treatment. **f** The effect of E2F1 knockdown on the protein levels of EZH2 and SUZ12 following immunoprecipitation with antibodies against EZH2 and SUZ12. shCtrl indicates cells transduced with a control vector. **g** Immunoblot analysis of EZH2–E2F1 interaction upon GSK126 treatment (1 μM). **h** The effect of GSK126 treatment on chromatin accessibility at the TSS of 306 cotarget genes of EZH2 and E2F1. **i** A model for the molecular mechanism of noncanonical association of EZH2 with E2F1 via chromatin remodeling.

To explore whether E2F1 modulates the methyltransferase activity of EZH2 and its association with SUZ12, we investigated the epigenetic consequences of E2F1 depletion in TNBC. The relatively higher expression of EZH2 and E2F1 (Supplementary Fig. 2e,f), along with their direct interaction (Fig. 2h) in TNBC, suggests a novel PRC2-independent molecular mechanism. The noncanonical function of EZH2 as a transcriptional coactivator was further investigated through H3K27me3 profiling. E2F1 perturbation by shE2F1 resulted in a global elevation in H3K27me3 levels (Fig. 5a and Supplementary Fig. 5a). About 24.4% among 1,630 H3K27me3-gained regions were located in promoter TSS regions (Fig. 5b). The transcription factor-binding motif analysis of regions with de novo gained H3K27me3 marks revealed significant enrichments of LSL1, SREBF2, HINFP, HIC1, PRDM1, E2F1, YY1 and ZNF341 binding motifs (Fig. 5c and Supplementary Fig. 5b). Most H3K27me3-enriched regions following E2F1 knockdown were colocalized with EZH2 and E2F1 peaks (Fig. 5d). Since H3K27me3 marks are associated with transcriptionally repressive states and closed chromatin, the impact of increased H3K27me3 levels on chromatin accessibility was analyzed. As expected, regions with H3K27me3 marks gained by E2F1 knockdown exhibited lower chromatin accessibility (Fig. 5e). These regions include the *CCNB1* and *RRM2* locus, for example (Supplementary Fig. 5c). Furthermore, E2F1 knockdown resulted in increased H3K27me3 accumulation, primarily at noncanonical EZH2 peaks, while no marked changes were observed at canonical EZH2 peaks (Supplementary Fig. 5d). These results are consistent with the observation that E2F1 interacts with EZH2 at the SANT2 domain critical for SUZ12 binding and enzymatic activity (Fig. 2i). The EZH2–E2F1 interaction may suppress the methyltransferase activity of EZH2 and inhibit SUZ12 association.

To determine the interaction between EZH2 and SUZ12 in the absence of E2F1, the effect of E2F1 knockdown was monitored. Even though E2F1 was previously known to activate EZH2 through the pRB–E2F pathway^17^, E2F1 depletion led to increased expression of EZH2 in our model, suggesting E2F1 has a context-specific role in EZH2 regulation. After E2F1 knockdown, the expression level of SUZ12 was also slightly increased and the interaction between EZH2 and SUZ12 seems to be enhanced (Fig. 5f). This suggests that the elevated levels of these proteins might have contributed to their strengthened interaction for the canonical PRC2 function. SUZ12 knockdown also induced global changes in chromatin accessibility, increasing accessibility in 913 regions and decreasing it in 3318 regions (Supplementary Fig. 6a). However, SUZ12 knockdown did not alter the chromatin structure at the promoter regions of co-downregulated genes by shEZH2 and shE2F1 (Supplementary Fig. 6b). Moreover, EZH2 inhibition led to reduced chromatin accessibility at noncanonical EZH2 peak regions, whereas SUZ12 knockdown induced only modest changes in these regions (Supplementary Fig. 6c). Canonical EZH2 peak regions exhibited very low chromatin accessibility and were not affected by EZH2 or SUZ12 knockdown (Supplementary Fig. 6d). Taken together, these results show that the interaction between EZH2 and E2F1 competes with EZH2–SUZ12 binding for PRC2 formation and provides a clue for explaining the noncanonical function of EZH2 in a context-dependent manner.

As shown in Fig. 1f, GSK126 treatment did not alter cell growth or the expression levels of EZH2 and SUZ12. Further analysis showed that inhibiting methyltransferase activity with GSK126 did not disrupt the interaction between EZH2 and E2F1 (Fig. 5g) and resulted in negligible changes in E2F1 binding profiles (Supplementary Fig. 7a,b). As predicted, GSK126 treatment had no effect on chromatin accessibility at cotarget regions of EZH2 and E2F1 (Fig. 5h) or on global transcription levels (Supplementary Fig. 7c). Collectively, these results indicate that inhibiting methyltransferase activity of EZH2 neither prevents its noncanonical roles with E2F1 nor blocks the cellular growth of TNBC.

## Discussion

EZH2 is highly correlated with breast cancer cell proliferation^45^. Several studies reported that EZH2, as a PRC2 component, contributes aggressive phenotype of TNBC by repressing tumor suppressor genes, such as *TIMP2*^46^ or *FOSB*^47^. Interestingly, TNBC shows the lowest levels of H3K27me3 among breast cancer types^19^ and exhibits drug resistance to EZH2 inhibitors^48,49^ despite the highest expression levels of EZH2. This paradox underscores the potential importance of noncanonical roles of EZH2 in TNBC. Therefore, this study aimed to thoroughly investigate the molecular mechanisms and physiological implications of EZH2 in TNBC, with a focus on its noncanonical function. In our previous research, we discovered a novel regulatory role of metal response element binding transcriptional factor 2 (MTF2) for PRC2 activity through genome-wide epigenetic profiling^50^. MTF2 is bound to *HOX* genes and is required for PRC2-mediated histone methylation. In a continuation of understanding PRC2-related regulation, this study applied multiomics approaches to examine the noncanonical roles of EZH2 and its regulatory mechanism in TNBC.

The elevated expression of PRC2 components, except *EED*, was confirmed in breast cancers compared to normal tissues. Notably, only *EZH2* showed higher expression in TNBC groups than other breast cancer subtypes (Fig. 1a, b). Also, EZH2 and EED proteins were more highly expressed in samples from patients with TNBC than in non-TNBC samples (Fig. 1c). Furthermore, cell growth was more profoundly suppressed by EZH2 knockdown than by SUZ12 knockdown, and inhibition of methyltransferase activity did not impact cell proliferation (Fig.1d–f). These results imply the presence of noncanonical functions of EZH2 independent of PRC2 in TNBC. Genome-wide profiling and molecular analysis revealed that the transcription factor E2F1 directly interacted with EZH2 in TNBC (Fig. 2). Notably, E2F1 was the only interacting transcription factor obtained from mass spectrometry analysis (Fig. 2k), which is consistent with the potential binding candidate recognized by transcriptional regulatory network analysis using downregulated genes upon EZH2 knockdown (Fig. 2e) and by motif analysis using noncanonical EZH2 targets (Supplementary Fig. 2a).

E2F1 is a well-established master regulator of the cell cycle, controlling key processes such as cell cycle progression, apoptosis and the DNA damage response^51^. Overexpression of E2F1 has been linked to poor prognosis of breast cancer^52,53^. In TNBC, E2F1 is particularly implicated in promoting aggressive tumor characteristics through the upregulation of *CCNA2*, which regulates both the G1/S and the G2/M transition phases, and *POLD2*, which is crucial for DNA replication and repair^54,55^. While the interaction between E2F1 and EZH2 in the activation of oncogenic pathways has been observed in other cancers, their collaborative role in breast cancer, especially in TNBC, remains largely unexplored^56,57^. This led us to subsequently investigate the mechanism by which EZH2 functions in association with E2F1 in TNBC.

Remarkably, the EZH2–E2F1 complex was found to mechanistically modulate chromatin accessibility, thereby promoting cancer cell proliferation while inhibiting G2/M phase arrest and apoptosis in TNBC (Fig. 3). Although E2F1 is primarily recognized for its role in regulating the G1/S phase transition, it has been reported that STAT3-activated E2F1 can promote the G2/M phase transition by upregulating genes including *CDK1* and *CCNB1*^58^. Therefore, the observed G2/M phase arrest upon the disruption of the E2F1–EZH2 complex may be linked to the upregulation of G2/M phase-related genes, including *CCNB1*, indicating the noncanonical role for E2F1 in promoting TNBC progression (Fig. 4). Moreover, this EZH2–E2F1-driven chromatin remodeling represents a novel function, analogous to the ‘chromatin-tuning factor’ role identified by our group as the novel mechanistic role of FOXD2 in colorectal cancer^23^.

Since E2F1 was identified as a potential binding partner of EZH2 for its novel function in TNBC, the regulatory mechanisms of the EZH2–E2F1 complex were further assessed. Specifically, E2F1 suppresses H3K27me3 deposition and enhances chromatin accessibility at target loci, thereby facilitating EZH2’s function as a chromatin context-dependent transcriptional coactivator. Mechanistically, E2F1 competitively binds to the SANT2 domain of EZH2, crucial for maintaining PRC2’s structural integrity (Fig. 2i). Thus, E2F1 binding reduced the H3K27me3 deposition and induced chromatin remodeling in its target regions by hijacking EZH2 from SUZ12 (Fig. 5). In contrast to EZH2 knockdown, treatment with GSK126, an EZH2 methyltransferase inhibitor, showed no effect on E2F1 binding, chromatin accessibility or transcriptional levels in EZH2–E2F1 targets (Fig. 5g, h and Supplementary Fig. 7). These findings suggest that EZH2, in cooperation with E2F1, exerts a distinct role in TNBC progression, independent of its methyltransferase activity.

Overall, this study reveals a novel noncanonical function of EZH2 in promoting cell cycle progression, independent of PRC2 (Fig. 5i). Our research highlights the predominant role of EZH2 as a coactivator with E2F1 in TNBC. While the EZH2–E2F1 interaction has been observed in a couple of cancer types, such as adrenocortical carcinoma and prostate cancer cell^56,57^, its mechanistic role in TNBC has not been explored. Our study not only confirms this interaction in a TNBC-specific context but also elucidates how this complex facilitates chromatin accessibility and cell cycle progression in a PRC2-independent manner. Moreover, we introduce a new perspective on E2F1, typically recognized as a negative regulator of PRC2, by demonstrating its involvement in chromatin remodeling and transcriptional regulation. As previously shown^59^, EZH2 may mediate open chromatin conformation through interactions with other chromatin modifiers, such as SWI–SNF complexes and p300. The resistance of TNBC to EZH2 inhibitors, despite high EZH2 expression, presents a substantional challenge in cancer therapy. Our results suggest that this drug resistance may arise from the noncanonical functions of EZH2. Consequently, while it is still essential to explore the broader implications of the noncanonical activity of EZH2 in other cancer types, targeting the novel function of the EZH2–E2F1 complex, such as developing mutations to block their interaction, could provide a new therapeutic avenue for treating aggressive cancers resistant to current EZH2 inhibitors.

## Supplementary Information

Supplementary information

## Data Availability

The raw sequencing data and the processed data are available in the Gene Expression Omnibus (GEO) repository at https://www.ncbi.nlm.nih.gov/geo/ with the accession numbers GSE272536 https://www.ncbi.nlm.nih.gov/geo/query/acc.cgi?acc=GSE272536 (ATAC-seq), GSE272537 https://www.ncbi.nlm.nih.gov/geo/query/acc.cgi?acc=GSE272537 (ChIP-seq) and GSE272538 https://www.ncbi.nlm.nih.gov/geo/query/acc.cgi?acc=GSE272538 (RNA-seq). The raw sequencing data are also accessible at the Korean Nucleotide Archive (KoNA) at https://kbds.re.kr/ with the accession number KAP240770. The public GEO datasets for EZH2 ChIP-seq (GSE223959) and H3K27me3 ChIP-seq (GSE77772) in MDA-MB-231 cells were downloaded and used for comparison. HMEC datasets were downloaded from the ENCODE Data Coordination Center (https://www.encodeproject.org/) under accession numbers ENCLB695ARL, ENCLB695ARM (EZH2-ChIP), ENCLB695AEL, ENCLB695AEM (H3K27me3-ChIP), ENCLB695AVW and ENCLB695AVX (Input).
