# Supplementary material for: Noncanonical association of EZH2 with E2F1 promotes tumor proliferation through chromatin remodeling
Source: Exp Mol Med. 2025 Dec 19;57(12):2886–97. doi: 10.1038/s12276-025-01603-0 (PMC12800308; doi:10.1038/s12276-025-01603-0)
Supplement: Supplementary file 1 — Supplementary information [file 12276_2025_1603_MOESM1_ESM.pdf]

Supplementary information

Non-canonical association of EZH2 with E2F1 promotes tumor proliferation through chromatin remodeling

Mijoung Yoo[1, #], Hyeonji Lee[1, #], Hyorim Park[1], Byunghee Kang[1], Hyo-Min Kim[2], Tae-Kyung Kim[1] and Tae-Young Roh[3,4,*]

[1]Department of Life Sciences, Pohang University of Science and Technology (POSTECH), Pohang 37673, Republic of Korea

[2]College of Pharmacy, Ewha Womans University, Seoul 03760, Republic of Korea

[3]Department of Life Sciences, Ewha Womans University, Seoul 03760, Republic of Korea

[4]Sysgenlab Inc., Pohang 37673, Republic of Korea

[#] These two authors contribute equally.

* To whom correspondence should be addressed. Tel: +82-2-3277-3890; Email: tyroh@ewha.ac.kr

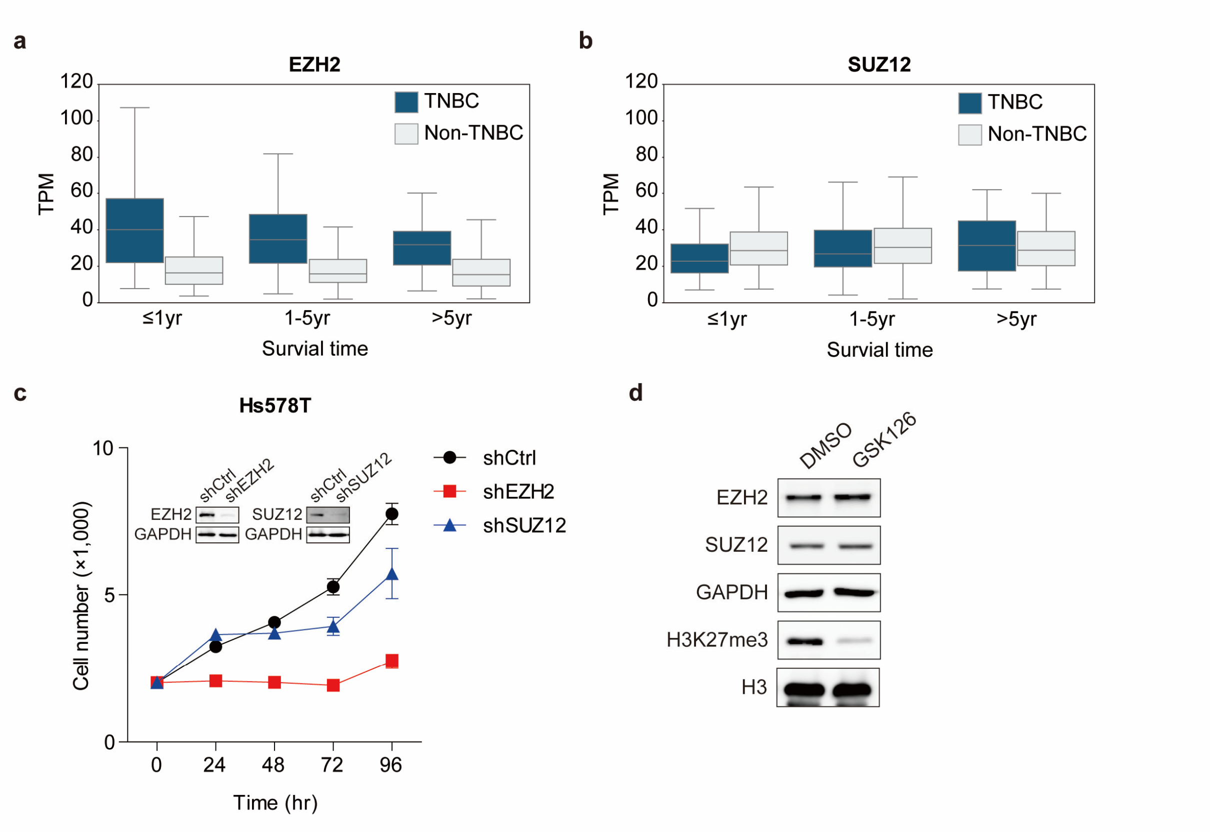

Supplementary Fig. 1. EZH2 is highly expressed in TNBC with low survival rates. a Comparative analysis of EZH2 expression levels in breast cancer patients. **b** Comparative analysis of SUZ12 expression levels in breast cancer patients. Patients were stratified into three survival groups: less than 1 year, 1–5 years, and greater than 5 years, based on overall survival duration. **c** The effect of EZH2 or SUZ12 knockdown on the cell growth in Hs578T cells. The inset figure shows the protein levels of EZH2 and SUZ12. **d** The effect of GSK126 (1 μM) on the expression levels of EZH2, SUZ12, and H3K27me3 in MDA-MB-231 cells.

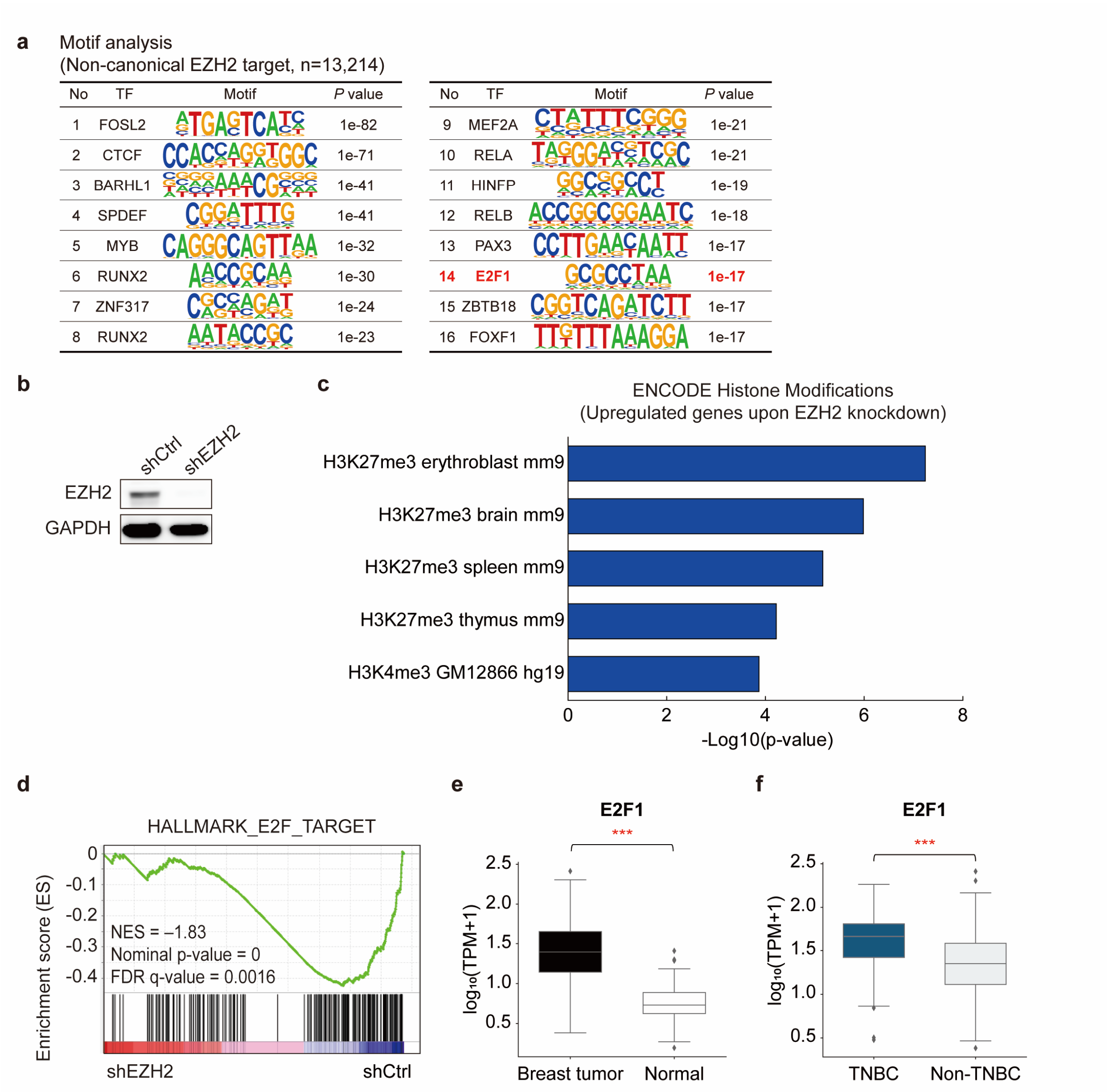

Supplementary Fig. 2. E2F1 is a potential factor for a novel EZH2 function in TNBC. a Top 16 motifs identified by the motif analysis using non-canonical EZH2 target regions (n=13,214). **b** The expression level of EZH2 before and after EZH2 knockdown. **c** GO analysis using the 952 co-upregulated genes after EZH2 knockdown. **d** GSEA analysis of E2F target genes based on downregulated genes upon EZH2 knockdown (n=1,391). **e** The expression levels of E2F1 in human breast tumors (n=1,116) vs. normal breast tissues (n=113) from the TCGA BRCA dataset. f The expression levels of E2F1 in TNBC groups (n=115) and non-TNBC groups (n=604) from the TCGA BRCA dataset. The statistical analyses were performed using a Mann-Whitney U test. All data represent mean ± SEM; ***P ≤ 0.001.

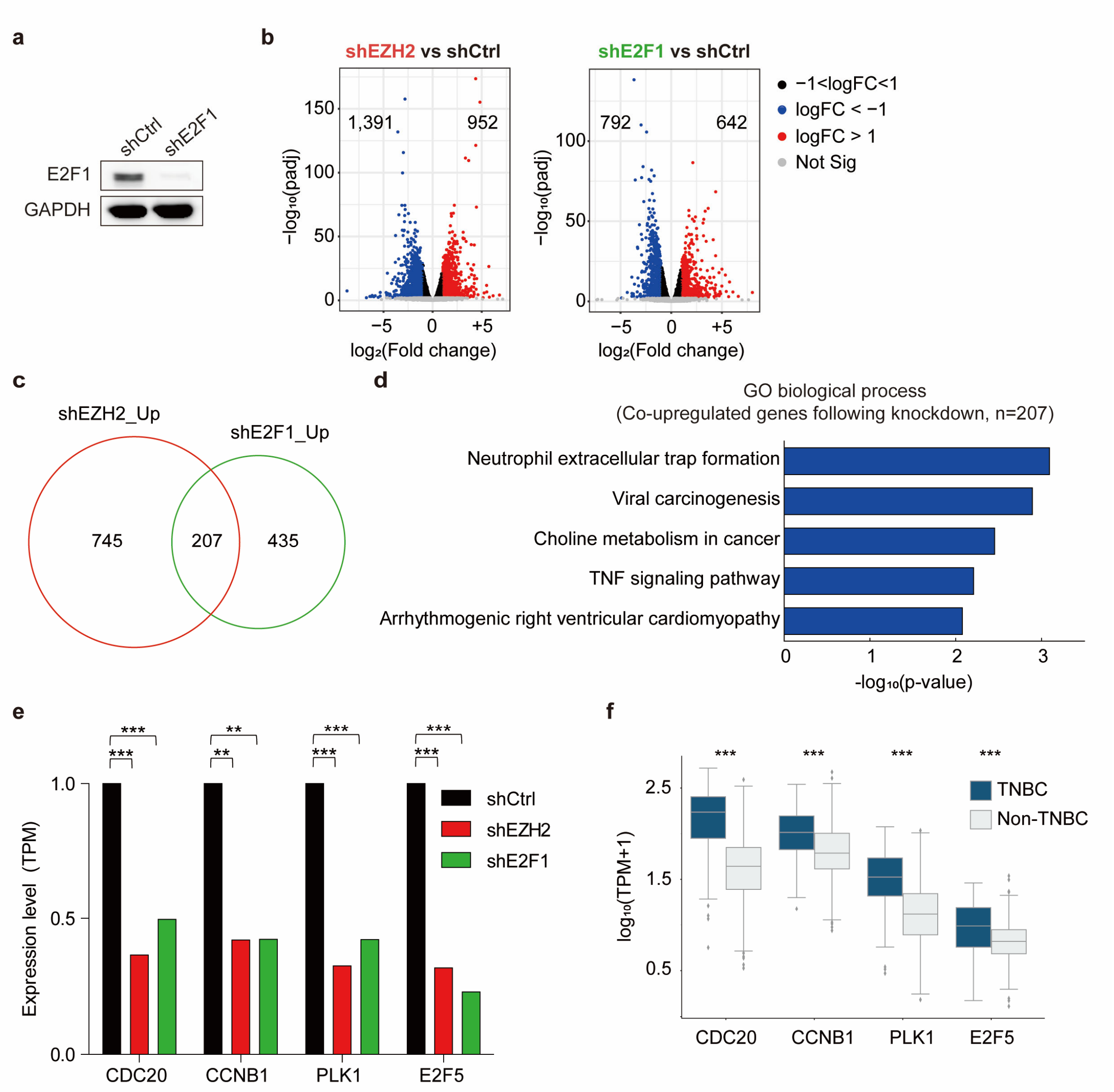

Supplementary Fig. 3. Biological roles by shEZH2 or shE2F1 were determined by comparative transcriptomic analysis. **a** The expression levels of E2F1 by shE2F1 treatment. **b** Gene expression changes induced by shEZH2 (left) and shE2F1 (right). The threshold of differentially expressed genes (DEGs) is set as adjusted p-value < 0.01, log (Fold change) > ±1. **c** Upregulated genes determined by shEZH2 and shE2F1 treatments. **d** GO analysis using the 207 co-upregulated genes. **e** Relative transcription levels of cell cycle-related genes following shEZH2 and shE2F1. **f** Transcription levels of cell cycle-related genes in TNBC groups (n=115) and non- TNBC groups (n=604) from the TCGA BRCA dataset. The y-axis shows log10 (TPM+1). All data represent mean ± SEM; *P< 0.05, **P ≤ 0.01, ***P ≤ 0.001, ns, not significant.

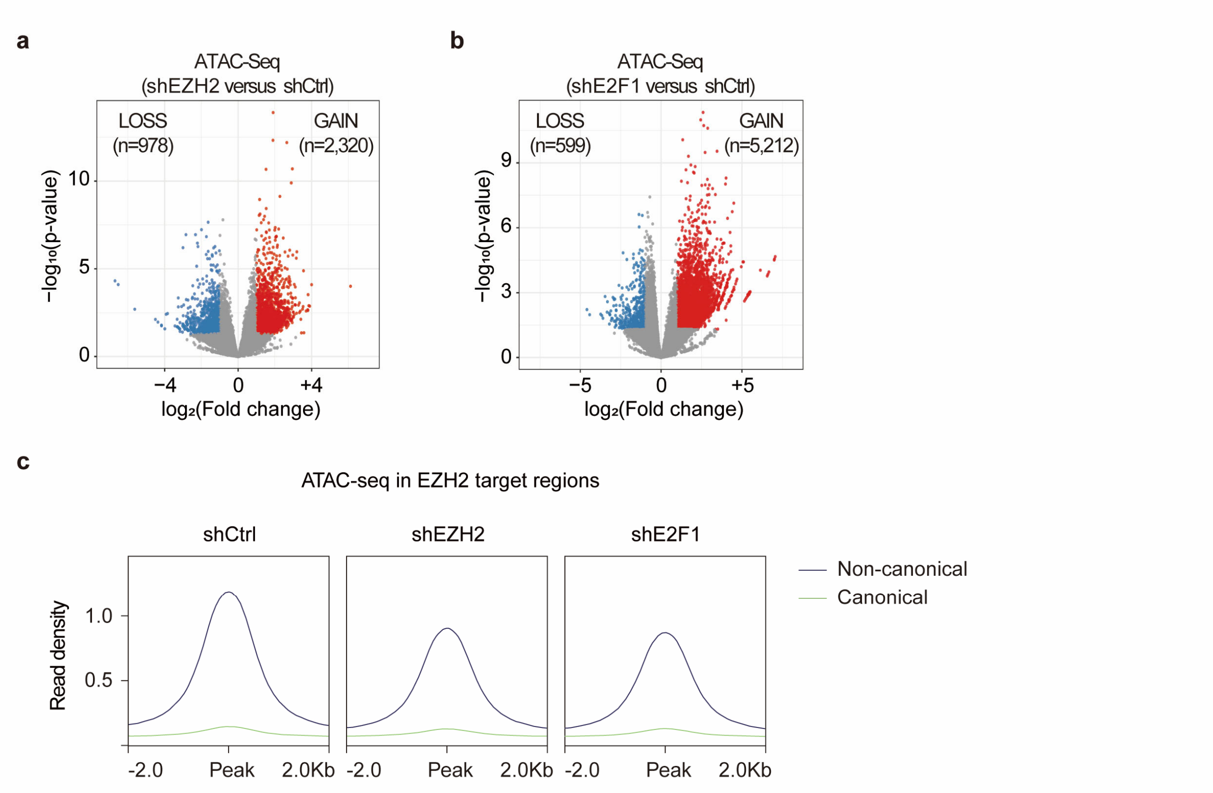

Supplementary Fig. 4. Chromatin accessibility was altered upon EZH2 and E2F1 depletion. a Changes in chromatin accessibility by shEZH2. **b** Changes in chromatin accessibility by shE2F1. The threshold of differentially accessible regions (DARs) is set as adjusted p-value < 0.05, log2 (Fold change) > ±1. **c** Effect of shEZH2 or shE2F1 on the global levels of chromatin accessibility around canonical and non-canonical EZH2 regions.

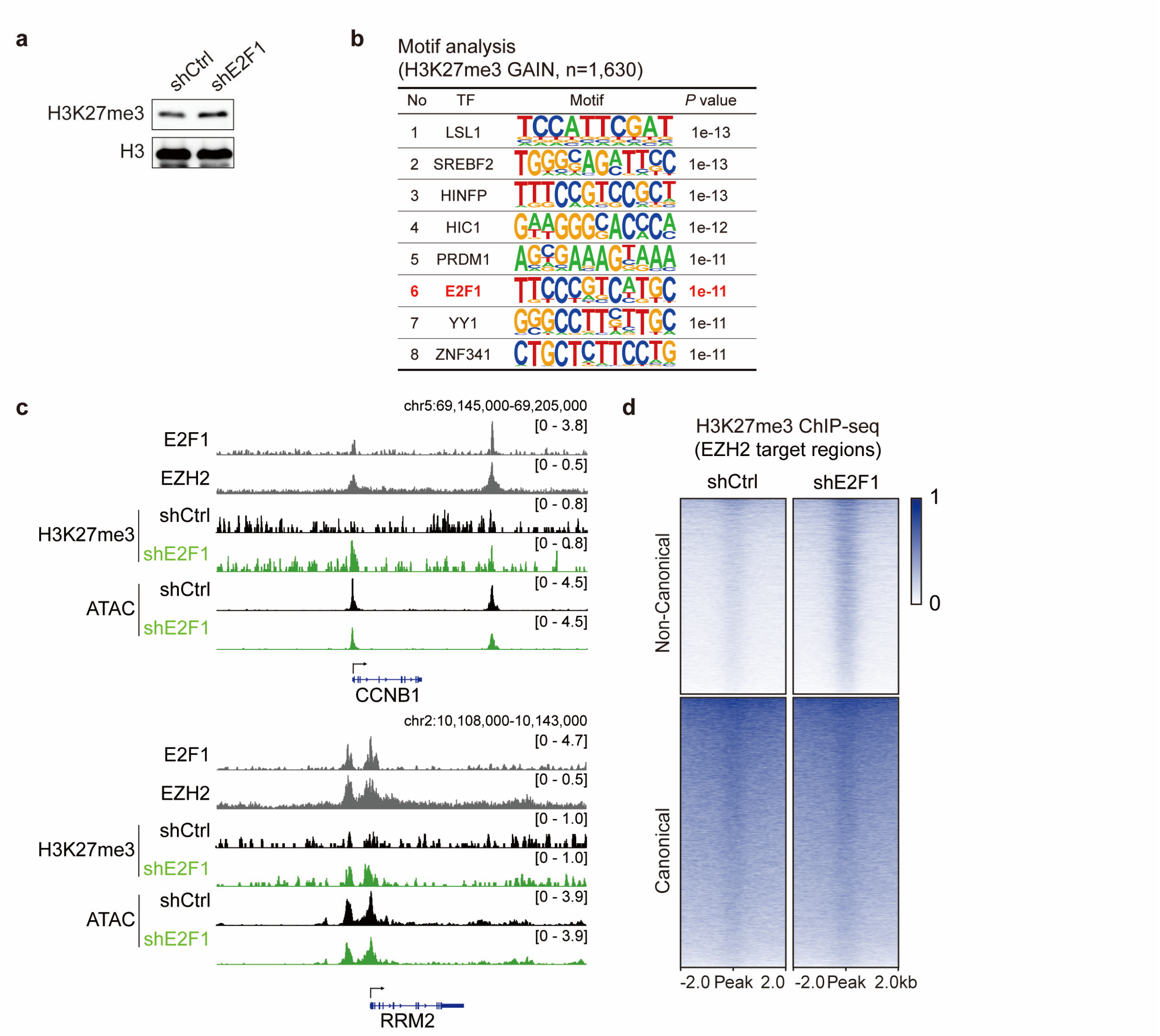

Supplementary Fig. 5. The altered H3K27me3 levels were examined by E2F1 knockdown. a The protein level of H3K27me3 upon E2F1 knockdown. **b** The top 8 motifs found at H3K27me3 GAIN regions (n=1,630). **c** Changes of H3K27me3 levels and chromatin accessibility by shE2F1 at *CCNB1* and *RRM2* regions. **d** Distribution of H3K27me3 marks affected by shE2F1 at EZH2+H3K27me3- regions (top, non-canonical regions) and EZH2+H3K27me3+ (bottom, canonical regions)

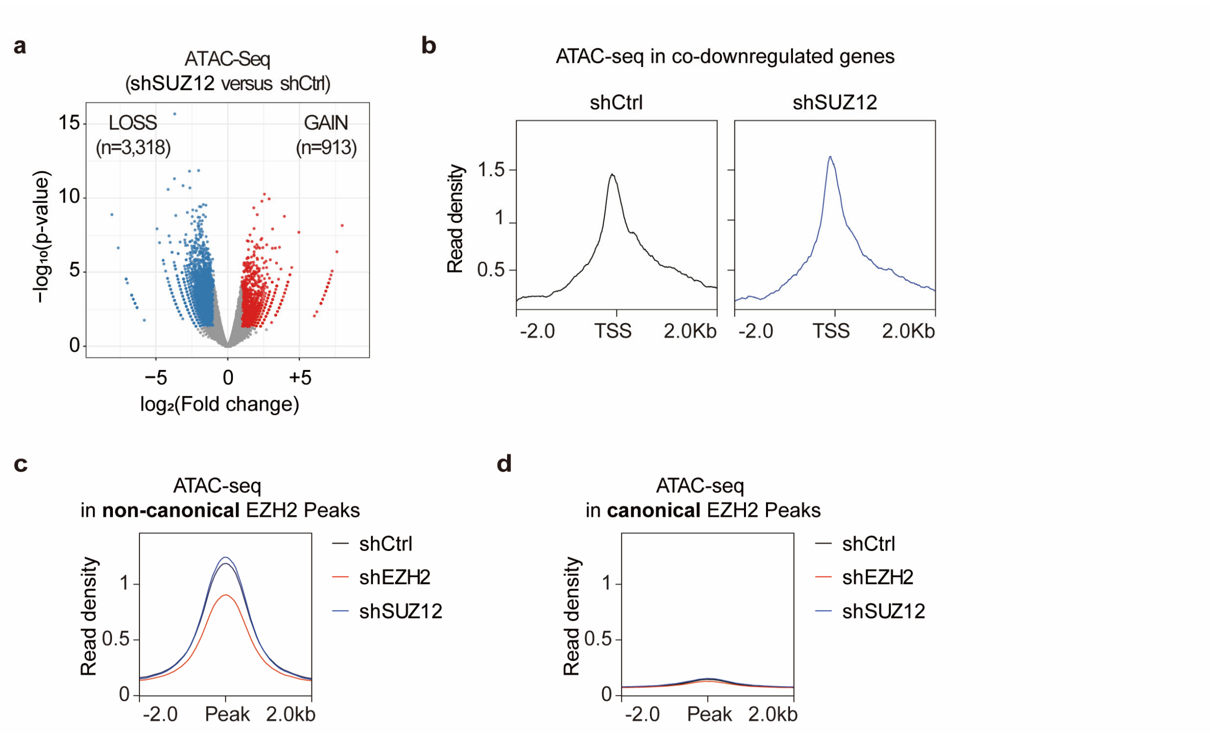

Supplementary Fig. 6. Chromatin accessibility was slightly changed upon SUZ12 depletion. a Changes in chromatin accessibility by shSUZ12. The threshold of differentially accessible regions (DARs) is set as p-value < 0.01, log2 (Fold change) > ±1. **b** Chromatin accessibility around TSS of the co-downregulated genes. **c** Chromatin accessibility around non-canonical regions. **d** Chromatin accessibility around canonical regions.

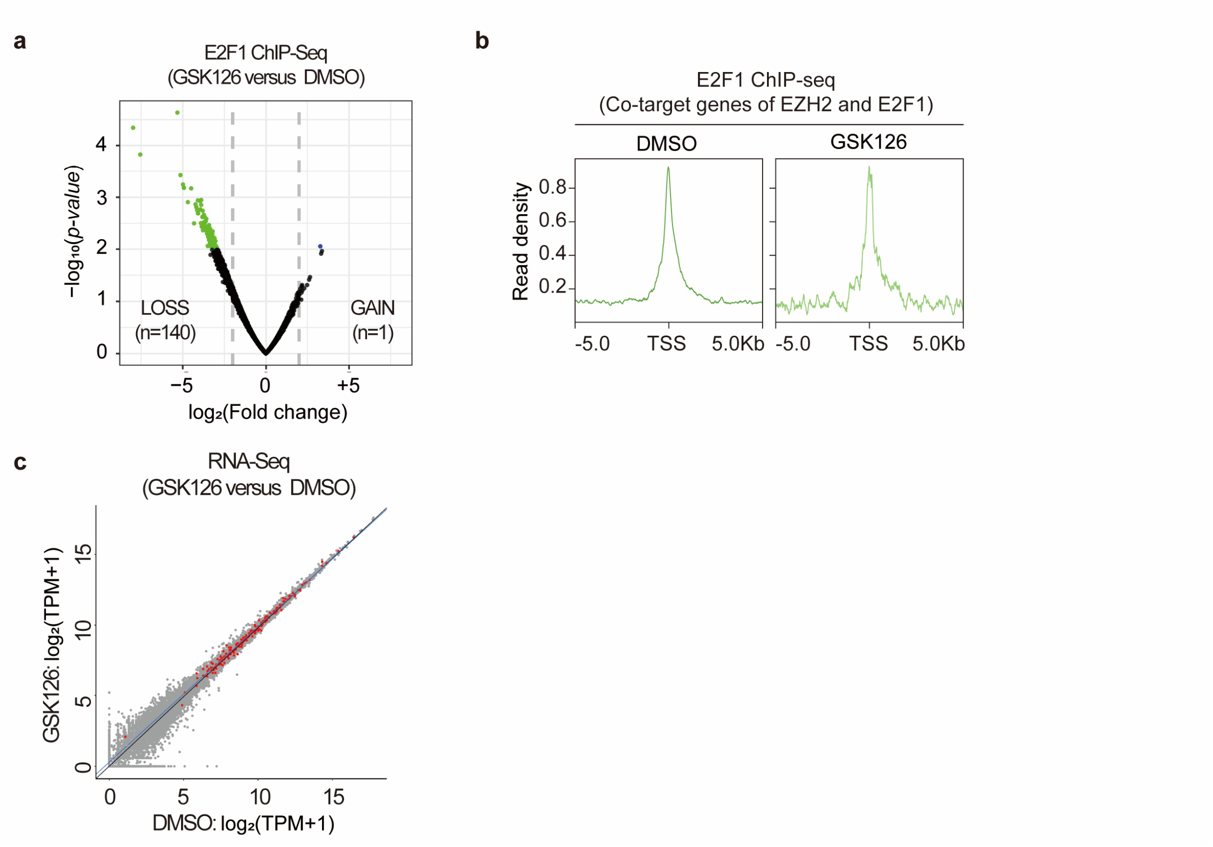

Supplementary Fig. 7. The non-canonical function of EZH2 was not impaired by the methyltransferase inhibitor. a Changes of E2F1 enrichments upon GSK126 treatment (1 μM). The threshold of DARs is set as p-value < 0.01, log (Fold change) > ±2. **b** Enrichment levels of E2F1 around 306 co-target genes of EZH2 and E2F1 upon GSK126 treatment (1 μM). **c** RNA-seq analysis for co-downregulated genes by shEZH2 and shE2F1 upon GSK126 treatment (1 μM).

Supplementary Table 1. Oligonucleotide sequences of shRNAs.

Name	Target Sequence
shEZH2	CGGCTCCTCTAACCATGTTTA
shE2F1	CAGGATGGATATGAGATGGGA
shSUZ12	GCTGACAATCAAATGAATCAT
shCtrl	TTCTCCGAACGTGTCACGT

Supplementary Table 2. Top 10% of proteins identified by mass spectrometry (MS) following immunoprecipitation (IP) of EZH2 or E2F1.

EZH2-associated proteins	LAMB3, RPL10, RUVBL2, SERBP1, P3H1, IGHA1, H2BC12, PKP1, UFD1, PPIB, GFAP, RPL12, RPS9, COL8A1, CRTAP, RPL8, KLC1, RPS20, HNRNPF, KIF5B, HNRNPH1, ARGLU1, PABPC4, CAVIN1, DSC3, LCN1, SPRR1A, RPL23A, LGALS7, H4C1, EED, SERPINB3, RPS15A, SPRR2G, RPS4X, HSP90AA1, PABPC1, PPIA, MYH9, PITRM1, PRDX2, KPLCE, RAB14, PCMT1, ARG1, FABP5, TUBA4B, ENO1, HSPB1, OPHN1, TUBB4A, RPS16, CTSD, RPS8, CAV2, FAF2, RPL10A, AZGP1, IGLC3, MUCL1, HBB, TGM3, IGKV1-17, H2AC11, DDX5, BLMH, IMPDH, ANXA2, TUBB6, HSPA8, ACTBL2, ITCH, SUZ12, TUBA4A, PLEC, SERPINB12, POTEF, FLG, CCN1, ACTB, PRSS3, JUP, PIP, RPL11, CAT, TUBA1C, GAPDH, DSC1, HSPA5, TUBB, TXN, CSTA, IGHG1, IGKC, CALML5, SRSF3, DSP, CASP14, CDSN, ALB, PRDX1, S100A8, EEF1A1, GGCT, SFPQ, S100A9, LYZ, NPLOC4, FLG2, S100A7, DSG1, EEF1A2, CPSF6, GRN, IGKV2D-24, E2F1, HRNR, NUDT21, RPS27A, CAV1, VCP, IGKV2-29, IMPDH1, IGHG3, IMPDH2
E2F1-associated proteins	RB1, DCD, A2M, CRYZL1, TRIM21, MAP4K4, NCL, RPL7, STRN, DDX5, EEF1A1, IGKV2-28, H1-2, IGLV2-33, RPL8, IGKV3-7, DSP, TRAK1, RPS2, NPLOC4, RPS6, RPL23A, RPS4X, RPL7A, PKM, KRT5, KRT10, RPS8, EEF2, KATNAL2, IMPDH2, LYZ, PABPC1, KRT73, RPS3A, TOP1, HSPA8, HSPA5, ACTB, H1-4, HSPA1A, UBB, VCP, GET4, HSPA2, KRT7, KRT19, HSP90AB1, LARP1, HNRNPM, ANXA2, KPRP, ENO1, RPL14, IGKV4-1, KRT18, RPLP0, SLC25A5, IMMT, CPSF6, GAPDH, KRT1, KRT6B, RPL29, LTF, ALB, LMNA, PUF60, KRT6A, KRT14, RPS9, IGHG1, BAG6, IGKV3-20, NONO, PLEC, LDHA, ARGLU1, HSPA9, FLNC, RPL4, KRT2, FUS, MYH10, MYH9, KRT9, FLNB, FAM111B, KIF5B, SF3B2, PABPC4, NUDT21, GRN, IGKV3D-20, OGT, AMOTL2, STRN4, AHNAK, EEF1G, FYCO1, RPL13, DDX3X, RPL18, RHOT1, CEP97, WRNIP1, RPS3, TUBG1, RPL6, SFPQ, PPP1R21, RBM14, STRIP1, SLMAP, HNRNPU, FLNA

Supplementary Table 3. List of antibodies.

Antibodies	Cat No.	Company
EZH2	5246S	Cell Signaling Technology
E2F1	sc-251	Santa Cruz
SUZ12	3737S	Cell Signaling Technology
EED	ab4469	Abcam
H3K27me3	39155	Active motif
Histone H3	ab1791	Abcam
GAPDH	ab9485	Abcam
Flag tag	F1804	Sigma Aldrich
HA tag	ab9110	Abcam
Goat Anti-Rabbit IgG H&L (HRP)	ab6721	Abcam
Rabbit Anti-Mouse IgG H&L (HRP)	ab6729	Abcam
Caspase 3	PA5-77887	Thermo Fisher Scientific
Ki67	14–5698-82	Thermo Fisher Scientific
Rabbit IgG	Sc-2027	Santa Cruz
